# Impact of instruction based on movie and TV series clips on EFL learners’ pragmatic competence: Speech acts in focus

**DOI:** 10.3389/fpsyg.2022.974757

**Published:** 2022-10-26

**Authors:** Fouad Rashid Omar, Özge Razı

**Affiliations:** ^1^Department of ELT, Faculty of Education, Cyprus International University, Nicosia, Turkey; ^2^Department of Foreign Languages Education, Faculty of Education, Education and Humanities Center, Cyprus International University, Nicosia, Turkey

**Keywords:** pragmatics, pragmatic competence, speech acts, movies and TV series, pragmatic instruction

## Abstract

This study attempts to investigate the role of movie and TV series clips in enhancing EFL learners’ pragmatic competence by utilizing an experimental design. The sample of the study was 42 students from the English language department at Cihan University-Duhok, Iraq. The experiment lasted one academic semester. The participants’ English language proficiency, as determined by an IELTS test sample, was intermediate, and then they were randomly split into two groups, namely experimental and control. Before and after the treatment, a Written Discourse Completion Test (WDCT) served as a pre-and post-test given to the two groups to assess statistically significant differences between them. The experimental group received direct instruction on request and suggestion speech acts via the presentation of the carefully chosen movie and TV series clips. In contrast, the control group was exposed to a minimal amount of pragmatics through printed texts. The findings demonstrated that the experimental group outperformed the control group. More precisely, the findings revealed that movie and TV series clips had a significant influence on learners’ production of requests and suggestions. Considering the above findings, the researchers propose EFL teachers apply movie and TV series clips to improve their Students’ pragmatic competence in class.

## Introduction

The emergence of communicative competence models (i.e., grammatical, sociolinguistic, discourse, and strategic competence) (Canale and Swain, 1980) marked a transformation in the perception of foreign or second language teaching and learning from merely mastering grammatical rules and memorizing vocabulary to learning how to use these elements functionally and socially. Thenceforth, pragmatic competence, which is defined as the ability to use the language appropriately and comprehend meaning in social situations, has emerged as a critical constituent of foreign or second language competency, different from the communicative competence models. More importantly, pragmatic teaching has seen a boost in popularity since pragmatics has started to acquire overt acknowledgment in communicative competence frameworks. This is recently proven by the fact that a substantial amount of literature including books and research has been published on the teaching of pragmatic competence and performance of different types of speech acts. Bachman and Palmer (1996) stress the importance of pragmatic competence by stating that it is the primary determinant of an individual’s overall language proficiency. Moreover, Kasper (1997) argues that foreign language instruction based on the coursebooks may help develop learners’ metalinguistic awareness but cannot improve their metapragmatic knowledge.

The ultimate goal of teaching a foreign or second language is to help learners communicate in a social context appropriately (Abdullah and Omar, 2020). Learners’ comprehension and production of speech acts can be negatively affected, even if they are fluent in the language and have excellent grammatical competency without receiving any instruction (Bardovi-Harlig and Mahan-Taylor, 2003; Alsuhaibani, 2022). In an environment where English is taught as a foreign language, the necessity for pragmatic teaching is necessary since learners are solely presented with the target language in classrooms. Rose and Kasper (2001); Yoshimi and Wang (2007), Alcón Soler and Martínez-Flor (2008), and Taguchi (2009) are examples of those authors who edited volumes based on empirical research that depicts instructional approaches and acquisition of pragmatic features in the classroom. Others are monograph-length studies, mentioning only the most recent research, that chronicle the process of pragmatic progress of language learners in institutional contexts (Abrams, 2014; Birjandi and Derakhshan, 2014; Alsmari, 2020; Alsuhaibani, 2022; Ziashahabi et al., 2020). All these publications underline the significance of teaching pragmatic competence and subsequently encourage EFL teachers to consider the most effective method to improve learners’ pragmatic competence. Therefore, the present study explores the impact of a method that is based on the use of movie and TV series clips on improving EFL learners’ pragmatic competence in the production of requests and suggestions. More precisely, the study’s objective is to address the following questions:

1. Does the use of movie and TV series clips help Kurdish EFL learners significantly enhance their pragmatic competence in making requests?
2. Does the use of movie and TV series clips help Kurdish EFL learners significantly enhance their pragmatic competence in making suggestions?

## Literature review

### Pragmatic competence

Although the majority of communicative competence models place a premium on the significance of pragmatic competence for performing successful interactions in a foreign or second language, it is a perplexing concept that has been hotly contested by a diverse range of professionals because of its connections to multiple different fields, including linguistics, sociolinguistics, psycholinguistics, and even cognition. Thus, there has been no wide agreement on defining the term pragmatics up to this point. Morris (1938) definition of pragmatics as “the discipline of the relations of signs to interpreters” was marked a revolutionary step. Consequently, Leech (1983) and Thomas (1983) expanded the notion of pragmatic competence by classifying it into two distinct categories: pragmalinguistic competence and sociopragmatic competence (as indicated before in the introductory section). According to LoCastro (2003), pragmatic competence is described as the ability to evaluate and interpret the meaning generated by the speaker and hearer via their joint acts. These acts involve both linguistic and non-linguistic signals and occur within the framework of socioculturally organized activities. It is more accurately defined by Yule (2017) as the meaning conveyed by a speaker or writer and perceived by a listener or reader. The most contemporary definition of pragmatic competence is provided by Ziashahabi et al. (2020), who indicate that pragmatics is a branch of linguistics concerned with the study of how individuals use language to communicate in a variety of social and cultural situations.

### Speech acts

Speech act theory is a branch of pragmatics that examines how words can be used to perform acts in addition to presenting the information. Austin (1975), in his book “How to Do Things With Words,” established the notion of speech act, which was further elaborated by J.R. Searle. The formation of speech acts is a tripartite process denoted by the terms locutionary, illocutionary, and perlocutionary. Locutionary act refers to “the utterance of a sentence with determinate sense and reference” (Levinson, 1983, p. 236). Any speech act activates certain spoken, syntactic, and semantic features of language to effectively achieve an illocutionary act that is the outcome of the articulated locutionary act’s real meaning. If the speaker says “Stop making that noise,” he or she is using a directive speech act that comprises certain phonological, syntactic, and semantic aspects to trigger an illocutionary act of warning to the listener, informing him or her not to keep making the unpleasant sounds. On the other hand, perlocutionary actions are distinct from both locutionary and illocutionary acts in that they pertain to the impact of an utterance act on the listener. Put it more simply, perlocution is concerned with how an act’s ramifications are affected by different illocutionary forces like convincing, acknowledging, cautioning, warning, and motivating.

According to Searle in Levinson (1983), there are five types of speech acts such as representatives, directive, commissive, expressive, and declaration. Yule (1996) provides an in-depth explanation of each of these speech acts. To begin, representative speech acts are those in which the speaker displays their belief in the truth or falsity of something. Propositions of fact, assertions, conclusions, and descriptions. For instance, when someone utters “The earth is flat,” the speaker expresses his or her views about the planet. He or she believes in the flattening of the earth. The speaker portrays the world in the way he or she perceives it to be. Directives are the types of speech acts used by speakers to compel others to perform something. They communicate the speaker’s meaning. They are commands, orders, requests, and suggestions that might be positive or negative. For example, when a speaker asks someone “Could you lend me a pen, please?”. Here the speaker is requesting an action from the listener. Commissives are those types of speech acts in which speakers express their commitment to perform an action in the future. They convey the speaker’s intention. They are promises, threats, refusals, and pledges. For instance, when a speaker says “I’ll be back,” he or she is promising the listener that he or she will return to them. Those types of speech acts that express how the speaker feels are known as expressives. They may represent psychological emotions such as pleasure, pain, likes, hates, joy, or sadness, and they can be expressed in many ways. Just as in this phrase “I’m really sorry,” this may be provoked by either the speaker or the hearer, but they are related to the speaker’s experience. Lastly, some other types of speech acts are referred to as declarations which, when delivered, have the potential to alter the destiny and world. For instance, when a priest declares, “I now pronounce you husband and wife,” the speaker must have a certain institutional position and in a particular setting to be able to properly conduct a proclamation.

The most common method of assessing learners’ pragmatic skills has been to examine their performance on speech acts, particularly when contrasted to that of native speakers. Using the appropriate speech act at the appropriate time and in the appropriate manner is believed to be part of a native speaker’s pragmatic competence, an instinctive knowledge that non-native speakers lack. Today, there seems to be an abundance of details and literature on how speakers realize the speech acts in certain settings, as well as predictions for how non-native speakers may deviate from these patterns in their speech productions. Unfortunately, the strategies that are used to construct these speech act utterances are mostly absent from the documentation on the subject (Cohen and Olshtain, 1993).

### Teaching pragmatics

When children learn their first language, it is often believed that they get explicit teaching from their parents. The parents do not fix their grammatical faults on a regular basis, nor do they teach them how to construct a proper sentence or how to pronounce a specific word. Over time, they will gain more confidence and the ability to produce semantically and syntactically accurate phrases and sentences (Alsuhaibani, 2022). When it comes to pragmatic mistakes, the parents are immediately engaged in rectifying the mistakes and teaching them how to use the language in a social context (Schmidt, 1993; Eun and Tadayoushi, 2006). Bardovi-Harlig (2001) and Kasper (2001) confirm the vast majority of language learners do not seem to learn the second language’s pragmatic elements on their initiative. Therefore, communicative competence methodologies have shifted the focus of foreign or second language teaching away from rote memorization of grammatical structures toward the pragmatic and social application of these formulas.

More importantly, the critical role of pragmatic knowledge in communicative competence has resulted in a substantial body of literature devoted to teaching foreign or second language pragmatics, especially speech acts. For the study, the researchers reviewed the literature on the influence of instruction on language learners’ pragmatic competence. Promisingly, meta-analysis and reviews of studies have shown that pragmatic instruction is important in developing learners’ pragmatic abilities (Rajabi and Farahian, 2013). Particularly, multiple classroom-based experimental research on learners’ pragmatic development has demonstrated that instruction intervention has a significant effect on learners’ pragmatic competence, even more so when learners are residing in a country where learners are merely exposed to the target language (Long, 1996; Eun and Tadayoushi, 2006; Belz, 2007; Takahashi, 2010a,b; Taguchi, 2015).

Furthermore, the researchers evaluated the literature on the most effective method of instruction to teach pragmatics in the classroom. There are two types of instruction, namely explicit and implicit which have been the central focus of arguments and research among scholars. However, there is a general consensus that explicit instruction is more effective than implicit instruction for teaching pragmatics, Alcón-Soler (2005) and Martinez-Flor (2005) showed the advantages of kinds of instructions in their study. On the other hand, Fazilatfar and Cheraghi (2013) study involved Iranian EFL university students and focused on investigating the effect of instruction on learners’ ability to produce compliments and comparing explicit instruction to implicit one. The participants were divided into three groups (explicit, implicit, and control). The explicit group received instruction using explicit feedback on the production of appropriate compliments, whereas the implicit group was given instruction based on implicit feedback. The findings of the study indicated that development occurred in both explicit and implicit groups, but the explicit group had a better performance than the implicit group.

Analogous results are reported in Ziashahabi et al. (2020). In their study, both the explicit and implicit groups benefit from instruction focused on producing speech acts and pragmatic competence improvement. The explicit group, on the other hand, outperformed the implicit group. As a result, explicit instruction was given precedence over implicit instruction as the learners who were exposed to the explicit instruction yielded a considerable development of pragmatic competence. Takahashi (2001) used a quasi-experimental (pre-test/post-test) design to examine the effect of four input enhancement conditions (explicit teaching, native speaker request comparison, native and non-native speaker request comparison, and reading comprehension) on the improvement of request strategies in Japanese EFL learners. The finding revealed that the explicit group performed better than the other three groups in terms of their utilization of the four request strategies. House (1996), Gaily (2014), and Li and Zhoumin (2019) found similar results in their study, stating that pragmatics cannot be efficiently learned without a pedagogical intervention.

As indicated above, researchers are becoming increasingly interested in examining the effects of the pedagogical intervention on second language learners’ pragmatic improvement in EFL/ESL settings. As Rose (2005) points out, the Noticing Hypothesis (Schmidt, 1990) justifies investigating the influence of teaching on learners’ pragmatic development. According to Schmidt, simple exposure to the target language is insufficient because there are pragmatic functions and pertinent situational variables many of which are taken for granted by learners and thus less likely to be noticed even after exposure for a lengthy amount of time. In contrast to Krashen (1985), who asserts that unconscious learning activities are superior to conscious learning activities and accountable for the lion’s share of second language output. Other scholars specialized in second language acquisition, such as Ellis (2008) and Schmidt (2012), claim that highlighting specific forms and directing learners’ notice to them may aid learners in language learning development. On the other hand, Rose (1994) emphasized the need for pragmatic consciousness-raising in pragmatic instruction. Abolfathiasl and Abdullah (2015) employed a pre-and post-test design to explore the effect of pragmatic consciousness-raising activities on Iranian EFL learners’ immediate and delayed execution of suggestions. For 8 weeks, the experimental group was subjected to pragmatic consciousness-raising treatment focused on the formulation of suggestions. The results showed that metapragmatic consciousness-raising significantly increased learners’ pragmatic performance and diversity of form-strategy application. Halenko and Jones (2011) also discovered that pragmatic awareness-raising had a positive influence on learners’ production of requests.

Another dimension of pragmatic instruction that should be taken into account by EFL teachers is the materials used in the classroom. Materials need to meet learners’ levels, needs, and interests, otherwise, they will get bored easily and reflect negatively on their performance. This is why EFL learners usually avoid studying a textbook. On the other hand, many EFL teachers put all blame on the textbook for lack of authenticity. Therefore, they recommend substituting textbooks with authentic materials such as movies, TV series, newspapers, magazines, etc., produced by native speakers for non-educational purposes (Nunan, 1988; Omar and Mekael, 2020). The primary objective of incorporating genuine resources into the classroom is to expose learners to as much actual language as necessary. According to Genhard (1996), authentic materials are essential for contextualizing language acquisition. Numerous studies have been undertaken on the influence of real materials on second or foreign language acquisition, but to the best of the researchers’ knowledge, only one study, as yet, has examined the effect of authentic materials on enhancing EFL learners’ pragmatic competence. This study was conducted by Abbasian et al. (2016) who used a quasi-experimental design. 60 EFL Iranian learners participated in the study. They were divided into two groups (experimental and control). The experimental group was exposed to authentic materials. The treatment consisted of 16 sessions of 90 min each. While the control group received standard pedagogical materials. The findings of the study found that the experimental group outperformed the control group. In other words, authentic materials had a great impact on developing learners’ pragmatic competence.

### Teaching pragmatics with movies and TV series

As previously stated, movies and TV series are considered an important part of authentic materials. According to the teaching experience of the researchers, a significant number of students have acquired English through watching movies and TV to the point where it is difficult to conceive how effectively and appropriately they utilized the language. Therefore, the role of movies and TV shows cannot be overlooked in the process of language learning. Teachers should encourage their learners to watch movies and TV shows to enhance their pragmatic competence since these media include authentic language. Heidari et al. (2020) argue that movies and TV series may substitute onerous textbooks in fostering learners’ pragmatic skills and teaching speech acts. Movies and TV shows have lately emerged as one of the richest resources accessible to EFL teachers, attracting the interest of a number of researchers in the field of second language acquisition. Various empirical research on the effectiveness of using authentic videos to teach speech acts in the classroom has been undertaken. In their study, Alerwi and Alzahrani (2020) suggest that the use of sitcoms may facilitate the learning of the speech acts of request, refusal, apologies, and compliment response by learners.

A study done by Alsmari (2020) assessed the effects of metapragmatic instruction on Saudi female EFL Students’ pragmalinguistic and sociopragmatic aspects while generating a speech act of complaint. Her experiment included a sample of 62 students who were majoring in English as a second language., the experimental group was presented with video-driven extracts, while the control group was exclusively taught via the usual manner. The results of the study showed that the experimental group that got a video-driven technique outperformed the control group in terms of performance and generated the speech act of complaint that were more suitable than those produced by the control group. After receiving the intervention, the students became also more expressive and provided longer replies as opposed to the linguistically constrained responses they provided in the pre-test. The results of her study also revealed that The participants themselves improved their ability to identify linguistic forms of politeness in real-world contexts. Similar findings were revealed in research done by Iranmanesh and Darani (2018). The findings indicated that movies had a substantial impact on the acquisition of idiomatic and daily English expressions among Iranian EFL students, hence enhancing their English proficiency.

Moreover, research has shown that movies and television programs may improve Students’ speech comprehension. Rodgers and Webb (2017) investigated Japanese university Students’ comprehension by exposing them to ten 42-min episodes of an American TV show with and without subtitles. The findings indicated that episodes help learners improve their comprehension.

In a study conducted by Hashemian et al. (2016), 37 upper-intermediate learners, aged between 21 and 25, from an English institute in Isfahan, Iran, were divided into two groups, experimental and control, based on their Quick Oxford Placement Test (QOPT) scores. A variant of the discourse completion test (DCT) was utilized as a pre-and post-test to evaluate the request and apology strategies employed by the respondents before and after the instruction. Participants devoted 30–40 min every Saturday and Wednesday for 7 weeks to viewing and analyzing clips from the movie Before Sunset. The results of the study indicated that the students were able to apply various request and apology strategies after watching the movie.

From an empirical standpoint, the utilization of movies is also beneficial for enhancing EFL learners’ pragmatic awareness. For instance, Rylander et al. (2013) study found that the application of video snippets from TV shows and movies improved learners’ speech act recognition, realization, notice, and production. In a study, Damaiyanti (2016) explored the influence of movies on the conversational implicature of language learners and discovered that movies may contribute to the development of learners’ understanding of speech acts.

Eryılmaz and Darn (2005) argue that EFL teachers must raise their Students’ consciousness of non-verbal communication to be able to communicate naturally, confidently, and effectively in the target language. They also underlined the need for teachers to assist students in eliminating intercultural misunderstandings since non-verbal communication is a system comprised of a variety of elements that are often utilized in conjunction to facilitate expression. It is believed that body language, such as facial expressions, gestures, eye contact, proximity, and posture (Eryılmaz and Darn, 2005) as well as intonation and taking turns (Coulthard, 1985; Kato, 2000), is essential to the language-learning process because they help learners be conscious of communicating messages, attempting to avoid misunderstandings, and adapting to the target culture (Kalra, 2017).

The situational context of a conversation refers to the environment, time, and place, among other things, in which it takes place, as well as the interaction between the participants. According to Yule (2000), context is the physical setting in which a word is used. Several studies have shown that context may play a significant influence in deciding the verbal and non-verbal choices of interlocutors and aiding them in achieving their communication goals (de Kok, 2008; Nobrega, 2009; Song, 2010; Abrams, 2014; Garten et al., 2019). A study conducted by Ismaili (2013) found that clips extracted from movies and TV series had a greater bearing on providing an authentic real-life environment for the production of speech acts.

## Methodology

### Participants

The sample of the present study comprised 42 Kurdish EFL junior university students studying the English language at Cihan University in Duhok, Iraq. They were randomly divided into two groups: the experimental group (*n* = 21) and the control group (*n* = 21). The participants were aged 21–25 years. To ensure the homogeneity of the participants, an IELTS test sample consisting of four sections (Listening, Speaking, Reading, and Writing) was used before the treatment. Only those students who achieved an intermediate level on the IELTS test were selected for the study since this level is optimal for developing pragmatic competence (Kasper, 2001). It is worth mentioning that the sample did not have any prior experience studying abroad and they had not also been exposed to any kind of pragmatics course before taking part in this experiment; hence, the treatment was their first introduction to pragmatics.

### Instruments of the study

Several instruments were utilized to collect data for the study. First, an IELTS test sample was used before data collection to verify the homogeneity of the chosen samples and to determine their level of language proficiency. Second, a Written Discourse Completion Test (WDCT) developed by the researchers was administered as the pre-and post-test. The WDCT test consisted of a total number of 10 scenarios concentrating on two kinds of speech acts; request and suggestion, which are most frequently utilized in every language and culture of the world. Each scenario was based on a situation in which the participants made a request and suggestion to someone in their family, social, or academic life. Such scenarios were also used to ensure that the data was as much authentic as possible. To validate the test, the test was sent via e-mail to five university professors whose specialization was pragmatics and second language acquisition from Iraq, Turkey, Cyprus, and India. They examined the clarity, precision, and appropriateness of the items of the instrument. The test was then piloted with a sample of 30 students from Knowledge University in Erbil to determine its reliability, which was discovered to be 0.821 utilizing Cronbach’s alpha. This score indicates that the items of the instrument have high internal consistency. The third and final instrument used in this study was Taguchi (2011) rating scale of appropriateness which included a comprehensive depiction of pragmatic and grammatical elements as a scoring system for evaluating the students’ responses to producing the targeted speech acts. Moreover, Taguchi (2011) appropriateness rating scale system consists of five points ranging from “Very poor” (1) to “Excellent” (5), as shown in Table 1.

**TABLE 1:** Taguchi’s (2011) rating scale. Reproduced from Taguchi (2011), with permission from John Benjamins Publishing Company.

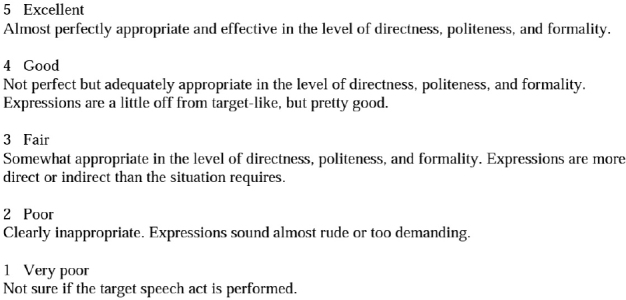

### Procedures

To gather the necessary data for examining the hypotheses under scrutiny, several procedures were followed. At first, an IELTS test sample comprising all four sections (Listening, Speaking, Reading, and Writing) was utilized to establish the homogeneity of the students. Following that, 42 students who attained intermediate level were chosen and then randomly assigned into two identical groups, one of which served as the experimental and the other as the control group. Each group consisted of 21 students.

The researchers devised the Written Discourse Completion Test (WDCT), which served as the pre-and post-test, and then sent it to five university professors with expertise in the field of pragmatics to check the validity of its items. After that, the WDCT test was piloted on a group of 23 students, from a different university, who had the same qualities as those participating in the main study for measuring the reliability of the WDCT test. Three qualified and experienced raters assessed and rated the data obtained through the pilot study.

Cronbach’s alpha was used to measure the reliability of the instrument. The value of reliability was acceptable which was 0.821. After the validity and reliability of the instrument were established, a pre-test was administered to both the experimental and control groups prior to the treatment. They had to answer the test within 30 min. It should be noted that the same techniques were used to assess and rate the responses of the participants on both the pre-test and the post-test. After that, the treatment process launched and lasted one academic semester of 12 weeks. There were two 60-min sessions every week, on Sundays and Thursdays.

The experimental group was presented with carefully chosen movie and TV series clips comprising varied instances of the targeted speech acts and were linguistically rich enough. The participants were exposed to four clips each session. These video excerpts were culled from various movies and TV series; specifically, *“Office Space,” “Friends,” “Fast and Furious 7,” “Frozen,” “Aladdin,” “Madagascar 3: Europe’s Most Wanted,” “The Blind Side,” “Sleeping Beauty,”* and *“Let’s Be Cops.”* The Participants engaged in pre-viewing, while-viewing, and post-viewing activities for each clip throughout the session.

The control group was only exposed to the conventional approach of pragmatics. They were only required to study pragmatics at the level specified in their textbook authored by Yule (2017), which was the bare minimum (i.e., defining and explaining speech acts requests, apologies, suggestions, refusal, and advice, and completing activities concerning these speech acts).

The post-test was administered to both groups after the treatment and the results of the pre-and post-test were compared. Once all of the data had been collected, it was thoroughly evaluated and analyzed using applicable statistical procedures from SPSS, such as the Independent samples *t*-test.

### Data analysis

The primary goal of this study was to investigate the influence of movie and TV series clips on Kurdish EFL learners’ pragmatic competence, especially on their ability to produce speech acts of requests and suggestions. Based on Taguchi (2011) rating scale, the Students’ responses to the pre-test and post-test were rated by three experienced researchers. Then the data obtained from the pre-and post-tests were statistically analyzed using SPSS version 24 statistical software. More precisely, paired-samples *t*-tests and independent-samples *t*-tests were employed to determine any significant differences in the pre-and post-test scores between the experimental and control groups.

## Results

### Effect of movie and TV series clips on Kurdish EFL learners’ production of requests

Concerning the first research question of whether clips from different movies and TV series have an impact on the participants’ performance in producing the speech act of request, the researchers used an independent sample *t*-test, which compares the mean between two variables (Blbas et al., 2022), to compare the mean scores of both pre-test and post-tests between the experimental and control groups, as shown in Table 2.

**TABLE 2:** Independent sample *t*-test between the experimental and control groups for pre-test (Request).

Pre-test	Speech act	Group	N	Mean	Std. deviation	*t*	*P*-value
	Request	Experimental	21	3.701	1.375	0.11	0.911
		Control	21	3.762	1.384		

**FIGURE 1:**
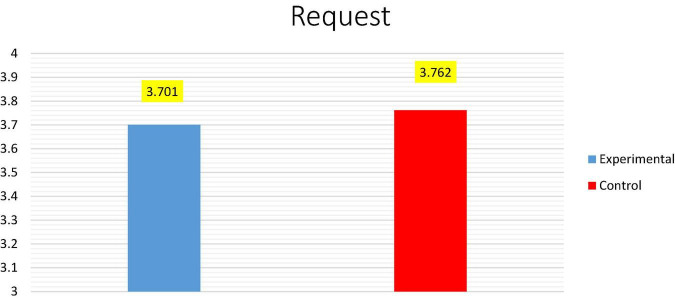
Shows the average between experimental and control groups for pre-test (Request).

Table 2 demonstrates that there is no statistically significant difference between the means of the experimental (*M* = 3.762; SD = 1.375) and control (*M* = 3.714; SD = 1.384) groups in the pre-test as their respective *P*-values (*t* = 0.11; *P* = 0.911) are greater than the significance threshold (*P* > 0.05). According to the findings, the level of performance shown by the participants was equivalent in some manner before the use of the instructional approaches as shown in Figure 1.

The post-test was administered to the two groups after the treatment had been administered for a full academic semester. An independent samples *t*-test was carried out to make a comparison between the post-tests concerning the production of the speech act of request, as shown in Table 3.

**TABLE 3:** Independent sample *t*-test between the experimental and control group for post-test (Request).

Post-test	Speech act	Group	N	Mean	Std. deviation	*t*	*P*-value
	Request	Experimental	21	6.524	1.401	4.244	0.001
		Control	21	4.429	1.777		

**FIGURE 2:**
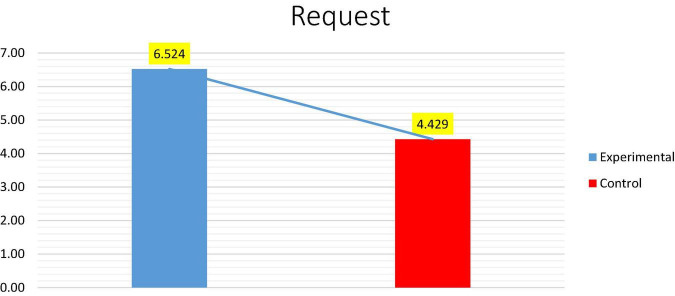
Shows the average between the experimental and control groups for post-test (Request).

Table 3 demonstrates that there is a statistically significant difference between the means of the experimental (*M* = 6.524; SD = 1.401) and control (*M* = 4.429; SD = 1.777) groups in the post-test, as its *P*-value (*t* = 4.244; *P* = 0.001) is less than the significance level (*P* < 0.05). The dramatic rise in the average score of the experimental group (*M* = 6.524) compared to the control group (*M* = 4.429), as depicted in Figure 2, suggests that the pragmatic instruction in the Language classrooms had a favorable influence on the pragmatic improvement of requests made by EFL students who participated in the pedagogical intervention.

To investigate differences among groups, a paired-samples *t*-test was used to compare the pre-test and post-test scores of the experimental and control groups before and after the treatment. According to the findings presented in Table 4, the performance of the participants in the experimental group substantially improved on the post-test, as indicated by a mean score of (*M* = 6.524, which represents a statistically significant improvement (*p* < 0.05) in comparison to their performance on the pre-test.

**TABLE 4:** Paired-samples *t*-test for experimental and control groups (Request).

Speech act	Group	Test	Mean	Std. deviation	*t*	*P*-value
Request	Experimental	Pre-test	3.701	1.375	11.60	0.000
		Post-test	6.524	1.401		
	Control	Pre-test	3.762	1.384	1.40	0.176
		Post-test	4.429	1.777		

**FIGURE 3:**
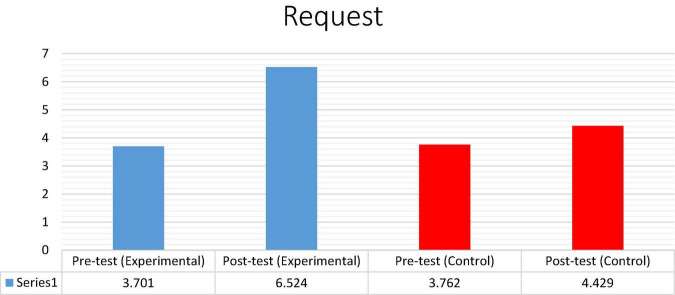
Shows the average between the pre-test and post-test for the experimental and control groups.

Whereas, as shown in Figure 3, the mean score of the control group on the post-test was marginally higher (*M* = 4.429) than on the pre-test (*M* = 3.762). This was due to the Students’ continued development as a consequence of traditional instruction.

### Effect of movie and TV series clips on Kurdish EFL learners’ production of suggests

**FIGURE 4:**
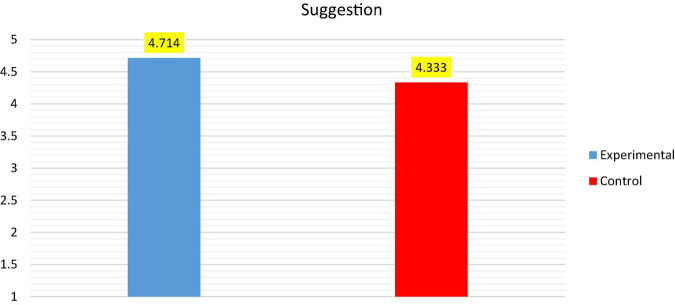
Shows the average between the experimental and control groups for pre-test (Suggestion).

To determine whether using snippets from movies and TV series has an effect on their aptitude for the production of suggestions in English, the mean scores of the pre-test were compared between the experimental and the control groups through an independent sample *t*-test, as shown in Table 5 and Figure 4.

**TABLE 5:** Independent sample *t*-test between experimental and control groups for pre-test (Suggestion).

Pre-test	Speech act	Group	N	Mean	Std. deviation	*t*	*P*-value
	Suggestion	Experimental	21	4.714	1.231	0.94	0.535
		Control	21	4.333	1.390		

Table 5 shows that there is no statistically significant difference between the mean scores of the experimental group (*M* = 4.714; SD = 1.232) and the control group (*M* = 4.333; SD = 1.390) prior to the intervention because the experimental group’s *P*-value [*t* = 0.94; *P* = 0.535 (>0.05)] is greater than the significant level of α = 0.05, respectively, indicating that the performance of both groups on the pre-test was similar. The mean score of the control group on the post-test was marginally higher (*M* = 5.048) than on the pre-test (*M* = 4.333), as shown in Figure 4.

**FIGURE 5:**
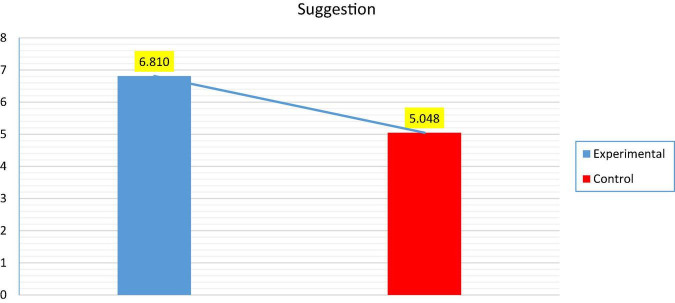
Shows the average between the experimental and control groups for post-test (Suggestion).

Table 6 demonstrates that the mean scores of students in the control group (*M* = 5.048; SD = 1.23) substantially varied from those of students who received movie and TV series clip-based pragmatic instruction (*M* = 6.810; SD = 1,250). Thus, there was a statistically significant difference between the mean scores of the experimental and control groups because the *P*-value [*t* = 4.654; *p* = 0.001 (<0.05)] is less than the significance level of α = 0.05 and the average of the experimental group (*M* = 6.810) is greater than the average of the control group (*M* = 5.048), as shown in Figure 5.

**TABLE 6:** Independent sample *t*-test between the experimental and control groups for post-test (Suggestion).

Post-test	Speech act	Group	N	Mean	Std. deviation	*t*	*P*-value
	Suggestion	Experimental	21	6.810	1.250	4.654	0.001
		Control	21	5.048	1.203		

**FIGURE 6:**
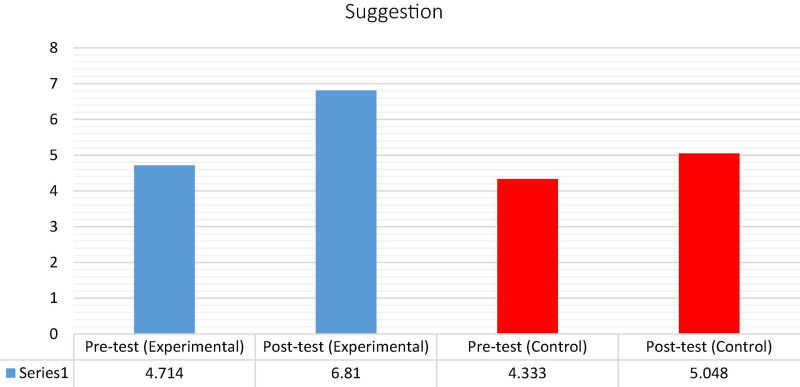
Shows the average between the pre-test and post-test for the experimental and control groups.

Using a paired-samples *t*-test, the effect of the intervention on the experimental group participants was investigated by comparing the means of the pre-test and post-test scores. As indicated in Table 7, the participants’ performance on the post-test drastically enhanced (*M* = 6.810) and was statistically significant (*p* = 0.001; *p* < 0.05) compared to their performance on the pre-test. By comparing the means of the pre-test and post-test scores, the paired-samples *t*-test was also used to analyze the effect of the traditional method of teaching suggestions on the participants in the control group. The findings indicated in Table 7 that the mean score on the post-test (*M* = 5.048) was noticeably higher than on the pre-test (*M* = 4.333), as shown in Figure 6.

**TABLE 7:** Paired-samples *t*-test for experimental and control groups (Suggestion).

Speech act	Group	Test	Mean	Std. deviation	*t*	*P*-value
Suggestion	Experimental	Pre-test	4.714	1.231	7.15	0.000
		Post-test	6.810	1.250		
	Control	Pre-test	4.333	1.390	2.85	0.010
		Post-test	5.048	1.23		

**FIGURE 7:**
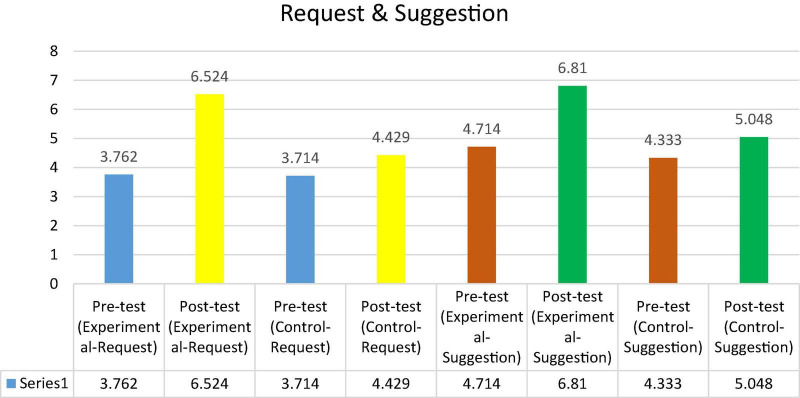
Shows the average between the pre-test and post-test for the experimental and control groups (Request and Suggestion).

To examine differences within groups, we used a paired-sample *t*-test to compare the pre- and post-tests in the experimental and control groups before and after the treatment. The findings in Table 8 demonstrate that the participants in the experimental group performed much better in the production of requests and suggestions on the post-test (*M* = 6.524; *M* = 6.810, respectively), a difference that was statistically significant *p*-value (*P* < 0.051) compared to the pre-test. The mean scores of the control group (*M* = 4.429; *M* = 5.048), on the other hand, marginally rose in the post-test, which may be ascribed to the participants’ continued growth as a consequence of the traditional approach of pragmatic aspects. In conclusion, the statistical difference between the two groups indicates the positive influence of implementing clips derived from movies and TV series in EFL classrooms to develop the EFL learners’ pragmatic competence and produce the speech acts of requests and suggestions. The mean scores of both pre-test and post-tests for the speech acts of request and suggestion are shown in Figure 7.

**TABLE 8:** Paired-samples *t*-test for the experimental and control groups.

Speech act	Group	Test	Mean	Std. deviation	*t*	*P*-value
**Request**	Experimental	Pre-test	3.762	1.375	11.60	0.000
		Post-test	6.524	1.401		
	Control	Pre-test	3.714	1.384	1.40	0.176
		Post-test	4.429	1.777		
**Suggestion**	Experimental	Pre-test	4.714	1.231	7.15	0.000
		Post-test	6.810	1.250		
	Control	Pre-test	4.333	1.390	2.85	0.010
		Post-test	5.048	1.203		

## Discussion

The objective of this study was to examine the influence of movie and TV series extracts on Kurdish EFL learners’ pragmatic competence, and specifically on their production of pragmatically acceptable and grammatically precise target-like requests and suggestions. Generally speaking, the findings of this study were promising as they revealed that the participants in the experimental group participants outperformed those in the control group. This study indicates that, when compared to traditional written texts, movie and television series clips may be considered preferable possibilities for teaching speech acts since they represent more realistic and communicative situations that occur in the actual world.

Considering the first and second research questions, the findings of the study found that there were significant differences between the experimental and control groups in the post-test. More specifically, the results revealed that the experimental group significantly improved their production of the speech act requests and suggestions after the intervention period. Consequently, it was found that the experimental group outperformed the control group. The findings of this study confirm previous research on the positive effect of instruction on learners’ development of pragmatics (Bardovi-Harlig, 2001; Alcón-Soler, 2005; Birjandi and Derakhshan, 2014; Rajabi and Farahian, 2013; Taguchi, 2015). Therefore, many believe that pragmatic instruction should be part of English classrooms just like grammar and vocabulary (Taguchi, 2015). In other words, the medium of instruction seems to play an integral role in learners’ acquisition of speech acts. Moreover, the findings of the study demonstrated that pragmatic aspects are teachable (Ishihara and Cohen, 2014).

The results of this study revealed that explicit instruction plays a great role in enhancing learners’ pragmatic competence and comprehending conversational implicatures correctly. Such a result is compatible with House (1996), Fazilatfar and Cheraghi (2013), and Ziashahabi et al. (2020). In their experimental study, it was even found that the participants who were exposed to the explicit instruction had not forgotten the materials they studied during the lessons.

The results of the study revealed that by teaching pragmatic elements through the use of consciousness-raising instruction, language learners produce and understand speech acts better than in the traditional method in which the learners only get some theoretical knowledge about the second language pragmatics. Such a result is in line with previous studies that involve teaching pragmatics through consciousness-raising instruction (Halenko and Jones, 2011; Abolfathiasl and Abdullah, 2015). Such a result support Schmidt (1990) Noticing Hypothesis, which argues that second language features cannot be acquired by learners without noticing, asserting that “while there is subliminal perception, there is no subliminal learning” (p. 26). Consciousness-raising tasks help learners to notice the norms, recognize, and process the differences in the production of requests and suggestions between English and Kurdish. In the process of noticing and comparing, learners eventually internalize L2 norms to become intake (Schmidt, 1993).

The results of the study are consistent with the findings of Abbasian et al. (2016), who investigated the influence of authentic materials on the pragmatic competence of EFL learners. The researchers found that providing authentic materials significantly improved the pragmatic skills of language learners.

The results of this study further proved that teaching pragmatics through movies and TV series clips is better than printed texts only. These results are in line with the findings of Heidari et al. (2020), who suggest that video clips derived from movies can be considered better alternatives for teaching speech acts compared to the traditional printed texts because these clips are authentic and reflect life-like, communicative events happening in the world.

## Conclusion

This study explored the impact of instruction by using movie and TV series snippets on EFL learners’ pragmatic competence. The results of the study indicate that teaching pragmatics using movie and TV series clips is effective and beneficial. The study shows that the production of speech acts such as requests and suggestions improved significantly with the use of explicit teaching incorporating movie and TV series clips. The results of the study show that when EFL learners are exposed to contextualized learning situations, their linguistic performance and pragmatic competence would improve drastically as a result of the rich input offered by real audio-visual resources. This is a strong invitation for teachers to reference movies and TV series when teaching pragmatics from a dull textbook to create an engaging classroom atmosphere. This study proposes a pragmatic intervention on English speech acts by using movie and TV series clips to increase Kurdish EFL Students’ knowledge of the pragmalinguistic and sociopragmatic variables related to the formulation of precise and appropriate requests and suggestions. Teachers and researchers should undertake more studies to explore the effect of movie and TV series clips on learners’ production of other speech acts as well as their comprehension, and investigate the Kurdish EFL learners’ views on this issue. Although the present study attempted to fill a vacuum in the area of pragmatics, the researchers must acknowledge some limitations. First, there were limitations regarding the sample. The small sample size makes it impossible to establish reliable generalizations. More research is needed to examine the results with greater sample size. Furthermore, all participants were intermediate-level university students. More research is needed to determine whether the same results would apply to students with varying levels of English proficiency. Second, because this was an experimental study, no qualitative data collection methods were used. Several qualitative and quantitative measures should be used to improve the validity of future studies. Finally, a written DCT was used as a pre-test and post-test to collect the data. Future research is required to use an oral DCT as a pre-test and post-test.

## Data availability statement

The datasets presented in this study can be found in online repositories. The names of the repository/repositories and accession number(s) can be found in the article/supplementary material.

## Ethics statement

The studies involving human participants were reviewed and approved by the Ethical Committee Board of Cyprus International University. The patients/participants provided their written informed consent to participate in this study.

## Author contributions

All authors listed have made a substantial, direct, and intellectual contribution to the work, and approved it for publication.
